# Use of ‘Aminogam Gel’ to fast the wound healing in dogs after the surgical curettage of injured penis

**DOI:** 10.1002/vms3.769

**Published:** 2022-03-01

**Authors:** Vincenzo Cicirelli, Gianluca Accogli, Michele Caira, Giovani M Lacalandra, Giulio Aiudi

**Affiliations:** ^1^ Department of Veterinary Medicine University of Bari ‘Aldo Moro’ Bari Italy

**Keywords:** Aminogam Gel, injured penis, canine penis, surgical curettage

## Abstract

**Background:**

Aminogam gel is used in human patients to accelerate the post‐surgical wound healing process of soft oral tissues (e.g. after teeth extraction or oral laser surgery). For this reason and because of the histological affinity between oral and genital mucosa, Aminogam Gel was applied on the dog's penile mucosa to evaluate wound healing after traumatic lesion.

**Objectives:**

This study aimed to compare conventional therapy (using only oral medications) to topic application of ‘Aminogam Gel’ in order to determine which is better to accelerate the healing process of canine penis injuries.

**Methods:**

For this study, 12 male dogs with an injured penis and traumatic paraphimosis were selected. All patients had traumatic penis injuries due to unsuccessful mating attempts and consequent trauma (continuous licking). The dogs underwent surgical curettage of necrotic areas. The animals were randomly divided into two groups: a control group treated with routine therapy and a group treated with Aminogam Gel as an adjuvant for the scarring process. We assessed wound status and tracked healing using the Bates‐Jensen Wound Assessment Tool.

**Results:**

Dogs treated with Aminogam Gel therapy healed faster than dogs treated with traditional therapy alone.

**Discussion:**

Aminogam Gel is a valid auxiliary drug to accelerate wound healing after penis surgery. This is especially important for breeding dogs, for whom rapid and complete healing of the penis is important for returning to normal reproductive activities.

## Graphical abstract

Aminogam Gel on traumatic lesions of the genital mucosa is an excellent therapeutic aid to be used in the canine species. The Aminogam Gel improves the healing of the mucosa injured.

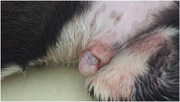

## 1 INTRODUCTION

In dogs, penile and foreskin abnormalities are very common pathologies and can be congenital or acquired (Cicirelli et al., 2021a; Pope et al., 1997; Root Kustritz, 2001; Tiftikcioglu et al., 2018). The treatment of these anomalies could require surgery or drug therapies depending on the damage severity (Birchard et al., 1998; Raheem et al., 2015; Leoci et al., 2019). When total necrosis (or extensive areas) of the penis occurs, amputation and surgical castration are recommended, as suggested by the literature on this topic (Boothe et al., 1993; Virasoro et al., 2015). Also, traumatic paraphimosis with penile necrosis is usually treated with amputation (Christie, 1981). As in human odontology, curettage of mucosal lesions is a good choice for wound healing in veterinary medicine (Carranza et al., 2002). Therefore, it is easy to understand that a drug capable of stimulating fibroblast proliferation can have a positive influence on the wound healing process (Ribatti, 2008). For this reason, the Aminogam Gel® (Errekappa, Italy) was tested. This gel used in human gingival medicine contains four amino acids (glycine, leucine, lysine and proline) and sodium hyaluronate. The effects of Aminogam Gel are as follows: proliferation of fibroblasts, deposition of collagen I and III (Mariggiò et al., 2009), production of growth factors involved in neo‐angiogenesis (vascular endothelial growth factor and transforming growth factor‐beta) (Romeo et al., 2014) and the expression of two inflammatory cytokines, interleukins 8 and 6, are involved in the recall of phagocytes and in the proliferation of keratinocytes (Colella et al., 2012). The literature indicates that Aminogam is used to accelerate the post‐surgical wound healing process of the soft oral tissues (after teeth extraction or oral laser surgery) (Cicirelli et al., 2017). Aminogam Gel promotes angiogenesis in vascular proliferation and has the capacity to induce, in human fibroblasts, the expression of an angiogenic cytokine, namely vascular endothelial growth factor. For all these reported reasons and because of the structural and histological affinity between oral and genital mucosa (Cook et al., 2005; Giovany et al., 2018; Markiewicz et al., 2007), Aminogam Gel was applied on the dog's penis mucosa to evaluate wound healing after removing the traumatic lesion (Mariggiò et al., 2009). This study aimed to compare conventional therapy (using only oral medications) to a new approach in order to determine which is better to assist and accelerate the healing process of penis injuries and make it more effective and comfortable for patients.

## 2 MATERIALS AND METHODS

### 2.1 Animals

For this study, 12 male dogs, some of which were breeding dogs, with an injured penis and traumatic paraphimosis were selected. The dogs were 24–90 months of age and weighed 12–26 kg. Good health status and low anaesthetic risk (American Society of Anesthesiology class 1) (Cicirelli et al., 2021b; Cicirelli et al., 2021c) was confirmed by routine blood tests (complete blood count and metabolic panel) and clinical examinations (including abdominal ultrasound). The dogs were housed with their owners, fed standard commercial dog food, and given water ad libitum. The dogs were routinely dewormed and vaccinated. The dogs selected for this study were divided into two groups: a control group (C group) treated with conventional post‐operative therapy and a group treated with the Aminogam Gel added topically on the lesion (A group).

### 2.2 Procedure

The animals were anaesthetised using the following protocol: pre‐medication with acepromazine at (Prequillan®; Fatro) at 0.02 ml/kg and metadone (Semfortan®; Eurovet Animal Health BV) 0.4 mg/kg), induction with propofol (Vetofol®; Esteve) at 4 mg/kg intravenously, and maintenance with isofloran (Isoflo®; Abbot). The patients were intubated with a tracheotube and placed in the dorsal position. Trichotomy and surgical scrubs were performed in the penile region. The surgical field was delimited using sterile surgical drapes fixed with a Backhaus towel clamp. At the beginning of the surgery, a urinary catheter was inserted to preserve the urethra. Surgical therapy consisted of elimination of necrotic tissue and surgical curettage of the affected part to allow vascularisation and increase healing speed. The surgeon made a tobacco pouch suture with the foreskin to protect the glans penis and resolve paraphimosis. Immediately after surgery, an Elizabethan collar was applied to prevent further self‐trauma. After the surgery, an antibiotic (amoxicillin, 25 mg/kg) and anti‐inflammatory therapy (robenacoxib, 0.5 mg/kg) were administered for 5 days. Additionally, a therapeutic gel, the Aminogam Gel, was applied until healing occurred (for 3 days within the preputium‐made purse in Figure 3 and for additional days spread on the penis surface) in the A group. No topical treatment was applied to the dogs in the C group. The patients at home were isolated from the bitches to avoid erections to prevent vascular damage or haematomas.

### 2.3 Bates‐Jensen wound assessment tool

We assessed the wound status and healing process using the Bates‐Jensen Wound Assessment Tool (BWAT) (Harris et al., 2010). The BWAT assesses the following nine items: size, depth, edges, necrotic tissue type, necrotic tissue amount, exudate amount, exudate type, granulation tissue and epithelialisation. This is important because the goal of wound care is to reduce wound severity. The total BWAT scores were divided into five severity categories: 0–8, healing; 9–19, minimal severity; 20–29, mild severity; 30–38, moderate severity and 39–45, extreme severity.

### 2.4 Data collection

All animals were monitored daily after surgery until all patient injuries had completely recovered (for a total of 14 days). The same surgeon monitored the wound status each day by collecting data for the BWAT.

### 2.5 Data analysis

The data collected using forms were entered and organized using a database created with excel software. Samples were grouped by Aminogam Treated and Control, and subsequently sorted by age (Cicirelli et al., 2021d). Statistical analysis was performed with SPSS Software using two‐way ANOVA and a post hoc Tukey‐Kramer test was applied to set the statistical significance of the data. Values were judged significant if *p* < 0.05 (Accogli et al., 2016; Cicirelli et al, 2021e). Data are presented as arithmetic means ± SEM.

## 3 RESULTS

**FIGURE 1:**
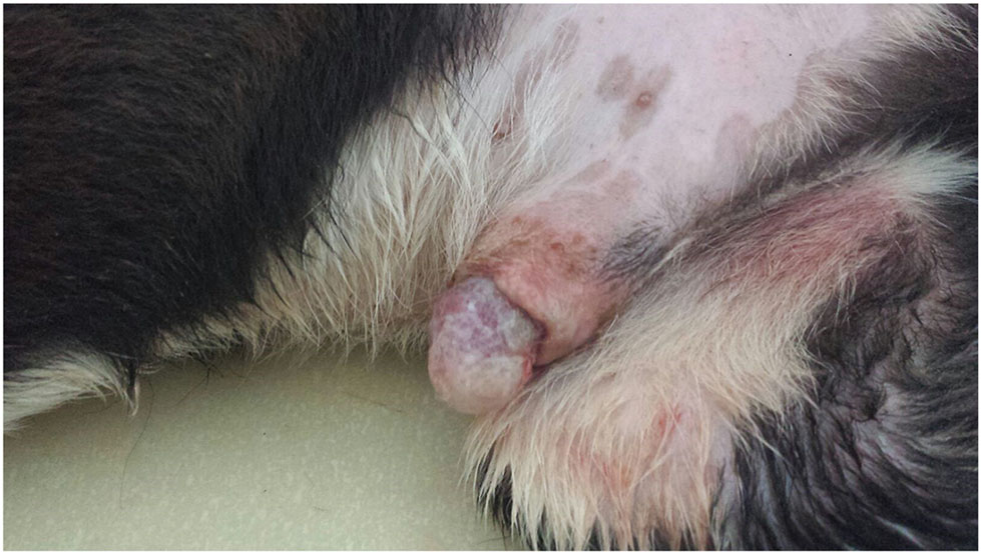
Dogs with an injured penis and consequent paraphimosis

**FIGURE 2:**
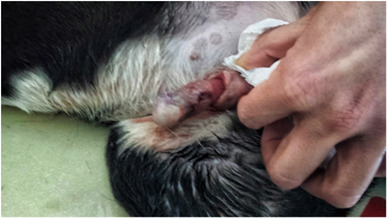
Dogs with an injured penis and consequent paraphimosis

**FIGURE 3:**
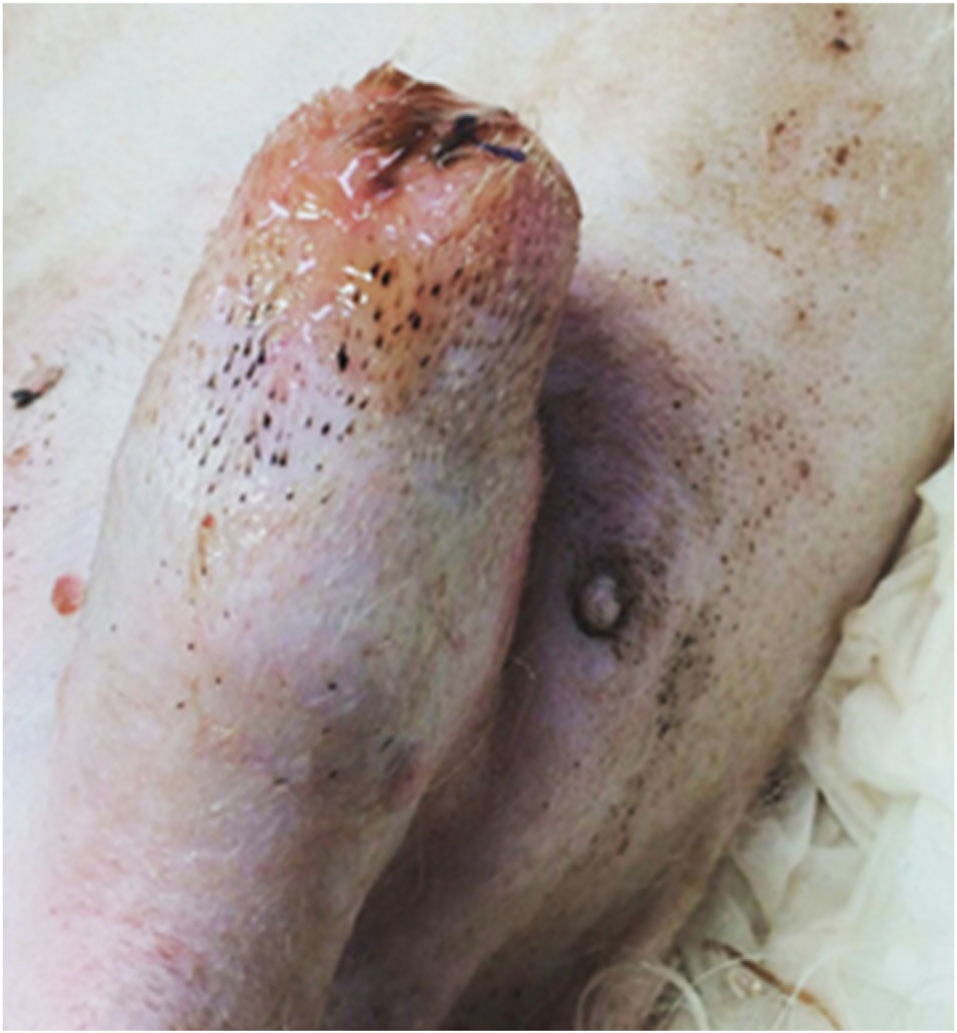
Amionogam gel in ‘the tobacco pouch suture’

**FIGURE 4:**
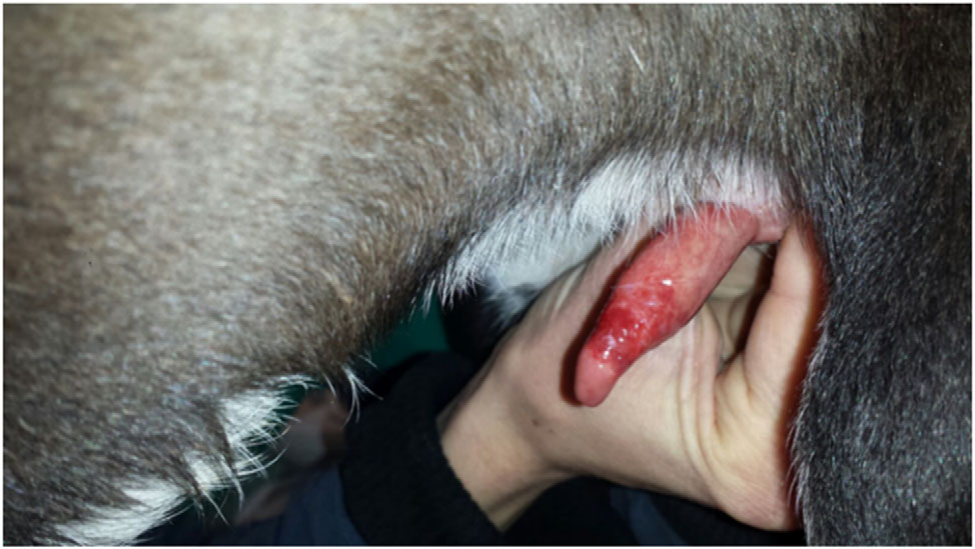
The healing of a patient recovered with the application of Aminogam Gel ointment

**FIGURE 5:**
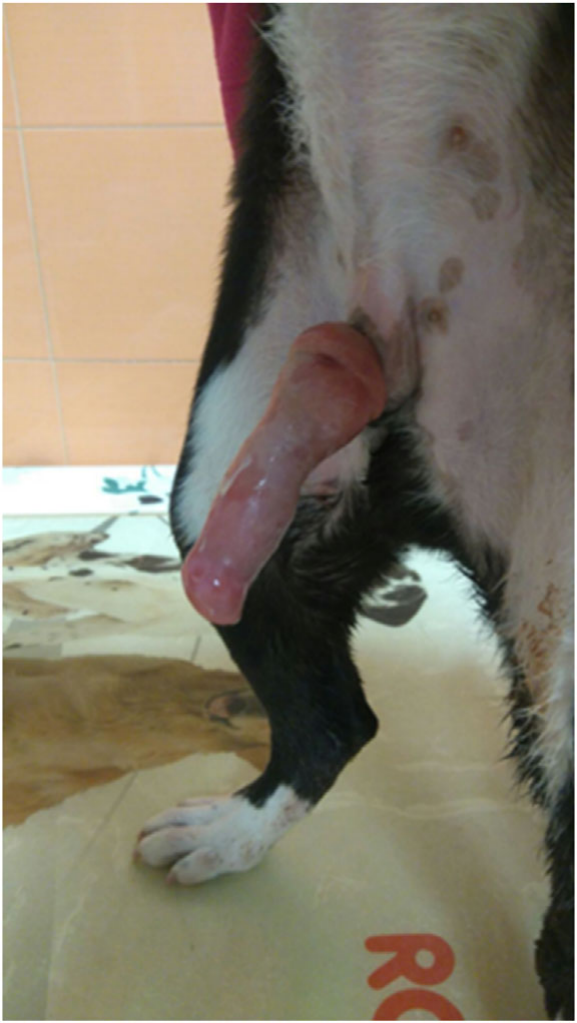
The healing of a patient recovered with the application of Aminogam Gel ointment

All the dogs selected in this study had penile lesions and traumatic paraphimosis due to unsuccessful mounting and licking of the genitals (Figures 1 and 2). Within 12 h after surgery, all the patients showed a clear clinical improvement and had started eating again. There were no post‐operative infections. The A group tolerated the administration of Aminogam Gel in the tobacco pouch of the foreskin (Figure 3). In this group, algic symptoms disappeared and attempts to lick the penis were rare after the first application of Aminogam Gel. The owners reported that the application of Aminogam Gel was simple and did not bother the dogs, either inside the tobacco pouch or on the surface of the penile mucosa. All owners went to the clinic daily to monitor their dog's penis until healing was complete (Figures 4 and 5).

All patients had similar lesions with a BWAT score higher than 42 (extreme severity). The dog was considered cured when his BWAT score was ≤8. Healing time was assessed by the veterinary team 2 weeks after surgery, based on daily checks. All pain symptoms and post‐operative discomfort gradually disappeared in both groups. In particular, in the C group, they disappeared from day 10 to day 11; in the A group, they disappeared from day 8 to day 9 (Graphic 1). In general, the healing process in both groups was similar until day 10. At the beginning of the experiment, the control group seemed to show a better response to the therapy, and the BWAT scores confirmed this. On day 8 of therapy, the values of both groups were the same; however, the treated group showed a subsequent improvement in scores and achieved healing point by day 11 (Graphic 1).

**GRAPHIC 1:**
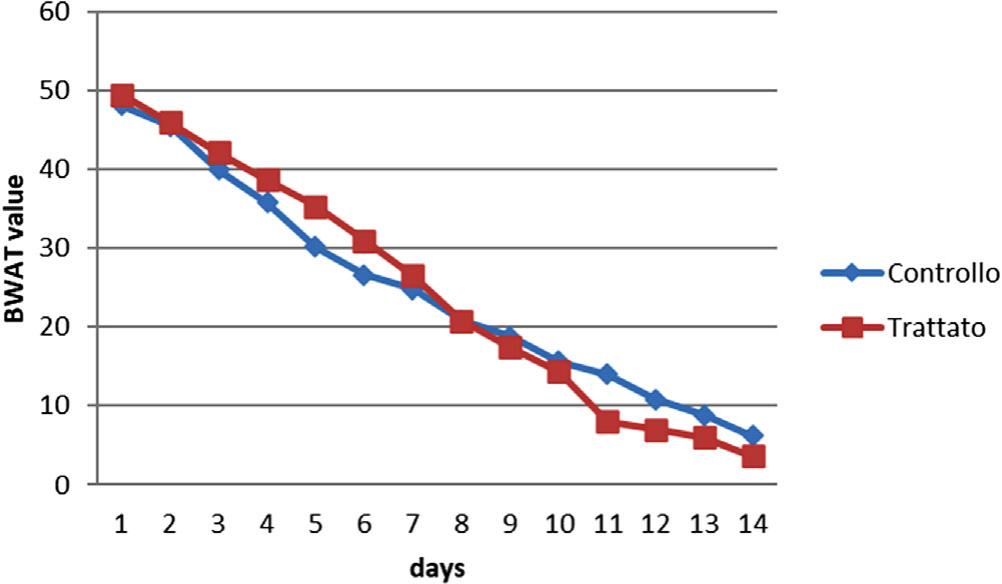
BWAT scale graphic describing the variation of patient's pain/discomfort until healing (BWAT = 8) in both control and treated groups

The dogs were divided into two groups according to age: young dogs under the age of 60 months and adult/old dogs over 60 months of age. The results in the Graphic 1 show that young patients in both the control and treated groups showed a higher score and consequently a better healing process. As shown in Graphic 2, the difference between the BWAT scores in young and adult/old patients was statistically significant. It is interesting to observe that the treated group showed significantly better BWAT scores in young compared with adult/old patients. In contrast, no differences were observed compared to the old ones (Graphics 3 and 4).

**GRAPHIC 2:**
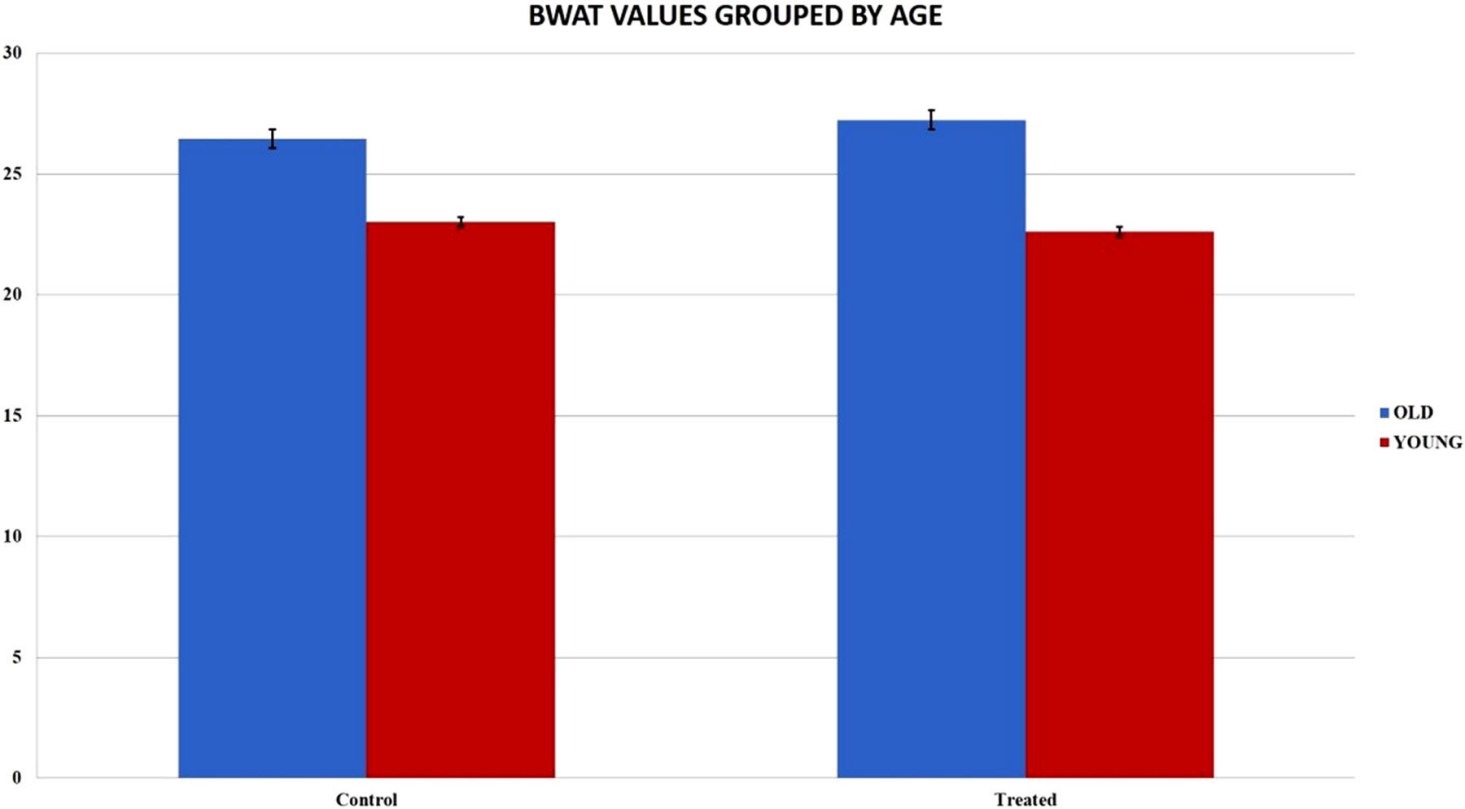
The difference between the BWAT values in young and old patients. It shows statistical significative differences; standard error bars were calculated

**GRAPHIC 3:**
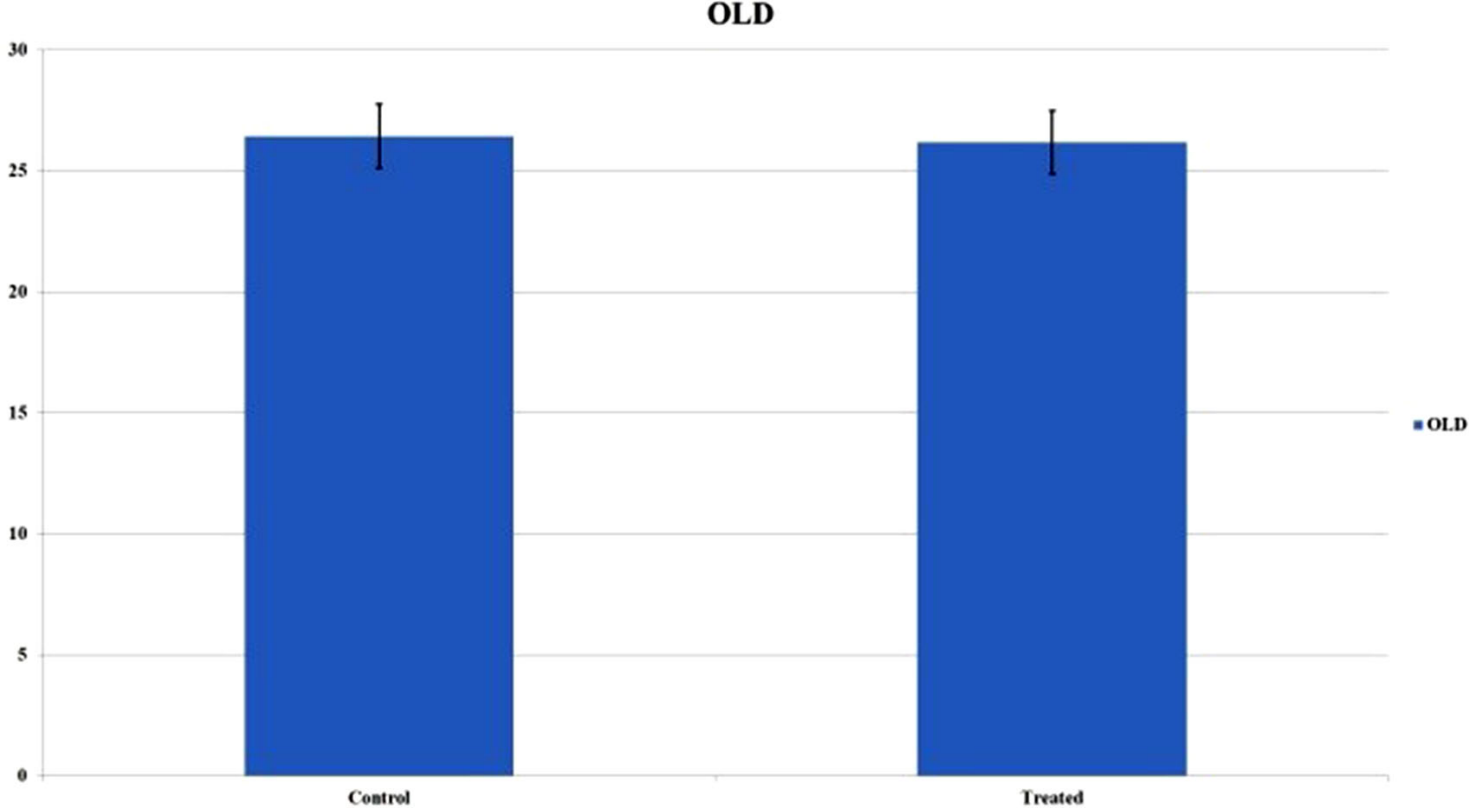
BWAT values in both groups: the treated group showed better score values in young patients and the differences were statistically substantial; standard error bars were calculated

**GRAPHIC 4:**
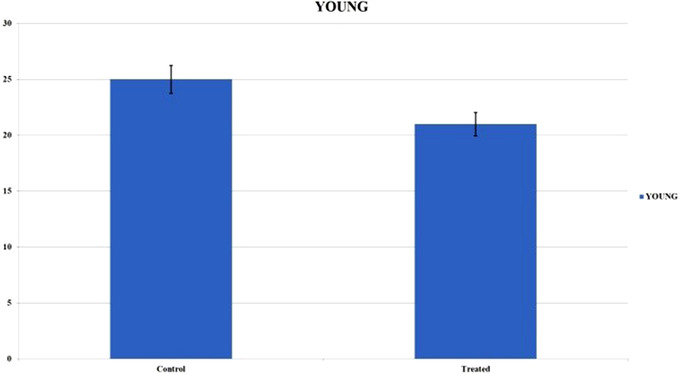
BWAT values in both groups: the treated group showed better score values in young patients and the differences were statistically substantial; standard error bars were calculated

## 4 DISCUSSION

This study aimed to combine a topical drug with therapy conventionally used for healing penile lesions in dogs. The aim was to accelerate the penis injury healing process and make it more effective and comfortable for patients. The idea for this work arose based on the similarity between oral tissue and genital mucosa, which is so strong that oral tissue is used to repair penis damage in humans (Giovany et al., 2018; Markiewicz et al., 2007). For this reason, a human gingival gel, Aminogam, was used in this study. All the dogs enrolled in this study had lesions on the penis mucous membrane due to traumatic couplings, which, together with continuous post‐traumatic licking, resulted in serious lesions on the penis of these animals, complicated by traumatic paraphimosis. In many cases, when the penis has large areas of necrosis, the recommended surgical therapy is penis amputation (Burrow et al., 2011). The veterinary team focused exclusively on conservative therapy using surgery and, consequently, medical therapy by mouth. In addition, topical therapy was used in the A group. In both groups, there was a complete healing of the penis. This study shows that dogs healed even without the application of topical products; however, the use of a topical drug accelerated healing. In human medicine, Aminogam Gel is also used in paediatric patients with positive compliance of young patients and parents as it is easy to handle, odourless, and tasteless and decreases algic symptoms by promoting oral hygiene behaviour and creating less inconvenience in feeding (Majorana & Cagetti, 2009). For this reason, it is a good choice as a topical treatment in veterinary medicine as this product does not have a strong odour or taste that can annoy patients. Colella et al. (2009) assessed the effect of Aminogam Gel in the treatment of oral mucosa. Aminogam Gel has also been used for oral mucositis, an inflammatory pathology characterised by localised erythema, ulcer formation, bleeding, and exudate formation that can occur as a complication in patients undergoing chemotherapy and radiotherapy (Vescovi & Manfredi, 2009). Taking into consideration these human studies, we tested Aminogam in veterinary medicine because it reduces the algic symptoms and its application between the glans and the skin of the foreskin prevented adhesions that could be an obstacle to lesion healing (Majorana & Cagetti, 2009). These studies have highlighted the antibacterial effect of Aminogam Gel (Colella et al., 2012). This could also reduce the amount of systemic antibiotic drugs administered to the patient, although a combination with oral antibiotic therapy is always recommended because it is a dirty wound (Singer, 2008; Simon et al., 2010). Our results demonstrated that young patients recovered better than adult/old patients. The use of Aminogam Gel significantly improved the speed of healing in both young and adult animals. Furthermore, as Graphic 1 shows, the improvement occurred during the second phase of healing, after day 8, which is considered the late healing phase (Nawar et al., 2005) when the most productive healing occurs. In the A group, the difference between young and adult animals was even more evident; which demonstrated the effectiveness of Aminogam Gel to accelerate healing. In the post‐operative period, the dog should not have erections because this can lead to the development of haematomas and vascular damage (Fowler et al., 1998). This is an important problem, especially in dogs with a good libido. Therefore, it is necessary to separate males from females that are in heat, and if this is insufficient, mild sedatives are recommended. In our patients, it was sufficient to isolate the males from the females without resorting to sedative drugs. The ultimate goal of reconstructive surgery is to preserve genitalia with normal function and appearance (Hunter et al., 2006), especially in breeding animals. For this reason, all dogs underwent spermiography to evaluate sperm parameters 45 days after surgery. All dogs were found to have sperm parameters within an acceptable range. Given our experience here verifying the short healing time in our patients, we believe the use of Aminogam Gel on traumatic lesions of the genital mucosa could be an excellent therapeutic aid to be used in the canine species. Glanuloplasty with oral mucosa graft following total glans penis amputation *Case Rep Urol*, Article 671303.

## ETHICS

This study was performed in accordance with the ethical guidelines of the Animal Welfare Committee. Institutional Review Board approval of the study was obtained from the University of Bari Aldo Moro with approval number 14/2020 (11/03/2020). Animal procedures were performed following the Directive 2010/63/Eu of the European Parliament (Italian DL 26/2014). Informed owner consent was obtained.

## CONFLICT OF INTEREST

The authors declare no conflict of interest.

## AUTHOR CONTRIBUTIONS

Vincenzo Cicirelli: Conceptualisation; investigation; methodology. Gianluca Accogli: Data curation. Michele Caira: Project administration; writing – review & editing. Giulio Aiudi: Supervision.

## FUNDING

This research received no external funding.

### PEER REVIEW

The peer review history for this article is available at https://publons.com/publon/10.1002/vms3.769.

## Data Availability

The data presented in this study are available on request from the corresponding author.
